# An Exploratory Retrospective Data Analysis Comparing the Outcomes of Selective Laser Trabeculoplasty and Argon Laser Trabeculoplasty in Patients with Open-Angle Glaucoma or Ocular Hypertension in Vienna, Austria, from the Year 2012 to 2022

**DOI:** 10.3390/medicina59122075

**Published:** 2023-11-24

**Authors:** Doreen Schmidl, Nikolaus Hommer, Anton Hommer

**Affiliations:** 1 Department of Clinical Pharmacology, Medical University of Vienna, 1090 Vienna, Austria; doreen.schmidl@meduniwien.ac.at (D.S.); nikolaus.hommer@oegk.at (N.H.); 2 Hommer Ophthalmology Institute, Albertgasse 39, 1080 Vienna, Austria; 3 Department of Ophthalmology, Hanusch Hospital, 1140 Vienna, Austria; 4 Department of Ophthalmology, Hera Hospital, 1090 Vienna, Austria

**Keywords:** glaucoma, selective laser trabeculoplasty (SLT), argon laser trabeculoplasty (ALT), retrospective, Vienna

## Abstract

*Background and Objectives*: The aim of the present study was to compare the short-term outcomes of selective laser trabeculoplasty (SLT) with argon laser trabeculoplasty (ALT) in patients with glaucoma in a real-world setting. *Materials and Methods*: The present study was conducted as a retrospective case–control study. The main outcome was the change in intraocular pressure (IOP) three months after laser surgery. In addition, the number of substances used for lowering of IOP and adverse events (AEs) were assessed. *Results*: Charts from 25 patients were included in the present study, of which 12 had received ALT and 13 SLT. In both groups, IOP significantly decreased from baseline values 6 weeks and 3 months after laser treatment (*p* < 0.01 vs. baseline at each timepoint for both groups). While after 6 weeks, no difference between groups was found, after 3 months, the decrease in IOP was significantly more pronounced in the SLT group (−26 ± 21% in the ALT group vs. −41 ± 14% in the SLT group, *p* = 0.018 between groups, ANOVA). Three months after laser treatment, the number of IOP-lowering substances used by each patient had decreased with no difference between groups (ALT: from 2.7 ± 0.8 to 2.3 ± 0.9 substances; SLT: from 1.8 ± 1.2 to 1.3 ± 1.1 substances, *p* = 0.386). Only a few AEs were observed. Two patients in the ALT and one patient in the SLT group required trabeculectomy within 1 year after laser treatment due to IOP decompensation. *Conclusions*: In the present study, SLT was at least as effective as ALT with fewer AEs and a similar reduction in concomitant IOP-lowering medication.

## 1. Introduction

Glaucoma is the leading cause of visual impairment and irreversible blindness worldwide. The mainstay of therapy is the lowering of intraocular pressure (IOP), which has been proven to slow down progression in the majority of cases [1,2].

The European Glaucoma Guidelines from the European Glaucoma Society (EGS) suggest starting treatment for newly diagnosed open-angle glaucoma or ocular hypertension using topical therapy or laser trabeculoplasty, while for more progressed cases initial surgery might be considered [3].

In clinical practice, in most cases, treatment is initiated with topical monotherapy. In cases where treatment is not well tolerated or IOP lowering is not sufficient, it should be switched to monotherapy from another substance class. If this again is not enough, dual therapy can be initiated. If after evaluation this is still not sufficient, substance classes and combinations should be switched again or a third substance might be added [3].

While all of these options lead to a multiple amount of drops that have to be administered by the patients, the EGS guidelines also suggest laser trabeculoplasty as an alternative to consider in all of the proposed therapeutic steps. Laser trabeculoplasty has been proven to be a safe procedure and its efficacy seems to be comparable to topical medication. In addition, there are no medication-associated side effects and there are also no compliance issues [4]. Laser trabeculoplasty reduces IOP by inducing biological changes in the trabecular meshwork, leading to an increase in aqueous outflow [2]. Therefore, laser trabeculoplasty has become more important in glaucoma and IOP management in recent years [2,4].

Argon laser trabeculoplasty (ALT) was introduced in 1979 [5,6]. Briefly, ALT uses a spot size of approximately 50 µm and spots have to be placed anterior at the border between the unpigmented and the pigmented area of the trabecular meshwork. Laser power has to be adjusted to the individual pigmentation of the treated eye [7]. However, scarring after treatment seems not to be limited to the trabecular meshwork but can be found up to the sclera [7]. Selective laser trabeculoplasty (SLT) is a procedure that has been developed more recently that has several advantages. Laser impulses are significantly shorter (approximately 3 ns vs. 0.1 s for ALT), which in turn reduces collateral damage at the surrounding tissue compared to ALT [7]. The SLT uses only 1% of the energy that is used in the ALT. Because SLT affects only the pigmented cells of the trabecular meshwork, there is no thermal damage at the level of non-pigmented cells or other structures of the trabecular meshwork. In addition, it is easier to operate [7]. In terms of IOP reduction, efficacy seems to be comparable between both methods [8,9]. However, with the ongoing development and improvement of devices, continuous comparison between different types of lasers remains necessary, as also suggested in a recent meta-analysis by Zhou et al. [5].

In Austria, the number of patients receiving laser trabeculoplasty for glaucoma treatment is constantly increasing. Several ophthalmologists are switching from ALT to SLT, due to the availability of devices that cause less collateral damage and are also more operator-friendly. The aim of the present retrospective study was to evaluate short-term patient outcomes in a real-world setting in a hospital (Hera Hospital, Vienna, Austria) and a private practice (Private Practice Dr. Hommer, Vienna, Austria) where this switch from ALT to SLT has already been performed. For this purpose, a case–control study was conducted where the eyes of patients that had received SLT were compared to the eyes of patients that were treated by ALT. The main outcome was a reduction in IOP after 3 months. Additionally, concomitant therapy was compared before and after laser treatment and adverse events (AEs) were assessed.

## 2. Materials and Methods

### 2.1. Study Design

The present retrospective study was conducted as a case–control study. Eyes that had received SLT were compared to patients that had received ALT. The study protocol was approved by the Ethics Committee of the Medical University of Vienna.

### 2.2. Patients and Sample Size Justification

All patients that were treated with ALT or SLT between 2012 and 2022 at the Department of Ophthalmology of the Hera Hospital or at the private practice of Dr. Hommer were evaluated, and those that met the inclusion criteria and none of the exclusion criteria were included in the analysis.

We included 25 data sets. The following precision could therefore be achieved in this exploratory study: The probability was 80 percent that the study would detect a treatment difference at a two-sided 0.05 significance level, if the true difference in IOP lowering efficacy between treatments was at least 4 mmHg. Smaller differences were deemed as not clinically relevant for the purpose of the present study.

### 2.3. Eligibility Criteria

In Table 1, in- and exclusion criteria for patients are presented. Patients were divided into two groups, namely those that had received ALT and those that received SLT. Only one eye per patient was included in the analysis. If both eyes of patients met the inclusion and none of the exclusion criteria, the eye with the higher IOP before the laser procedure was included. If IOP was equal, the right eye was included.

### 2.4. Types of Laser

SLT: SLT was performed with a frequency-doubled 532 nm Nd:YAG laser (YC-200 S plus, Nidek, Japan). The energy was set to 0.2–2.0 mJ depending on bubbles generated and then set just below that energy level, and the duration of a pulse was 3 ns. SLT was performed over 360° of the chamber angle.

ALT: A Coherent Novus 2000 (Coherent, Santa Clara, CA, USA) ALT laser with a wavelength of 488nm was used. ALT was performed with an energy of 1000mW or less, depending on the formation of bubbles (like SLT) and a pulse duration of 0.1 s over 360° of the chamber angle in single mode.

All laser procedures were performed by the same experienced physician (A.H.).

### 2.5. Statistical Analysis

Statistical analysis was performed using SPSS Statistics (Version 27.0, IBM Corp., Armonk, NY, USA). The reduction in IOP 3 months after laser treatment was the primary outcome. Descriptive statistics were applied for description of the study population and to report frequencies of AEs and percentages of patients that achieved IOP reduction of >20%. Chi-square test was used to assess differences in sex and glaucoma type. Paired *t*-tests were used to assess differences in IOP values between baseline and after 6 weeks or 3 months within groups, respectively. A repeated-measures ANOVA model was used to compare the time course in changes in IOP and number of IOP-lowering substances used between the two groups.

## 3. Results

Out of the patients’ records, 25 patients (16 female/9 male) who had received ALT or SLT treatment that met the inclusion and none of the exclusion criteria were identified. Overall, 12 eyes were treated with ALT and 13 received SLT treatment. The baseline characteristics are provided in Table 2. There was no difference regarding age, sex, glaucoma type or IOP at the timepoint of glaucoma diagnosis between the two groups. All patients had mild-to-moderate glaucoma according to the EGS staging criteria [3]. The most frequently reported systemic diagnosis was arterial hypertension.

**Figure 1:**
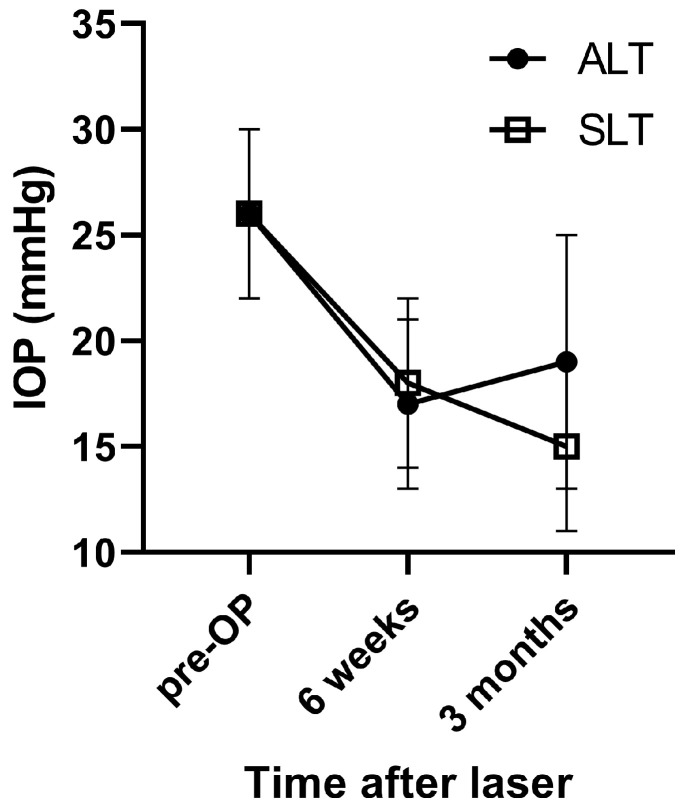
IOP values of the two study groups at different time points. Pre-OP values were obtained within two weeks before laser treatment and while patients were under topical therapy. Values are provided as mean ± SD.

In both groups, IOP significantly decreased from baseline values. In the ALT group, a decrease from 26 ± 4 mmHg to 17 ± 4 mmHg was seen after 6 weeks (*p* < 0.001) and to 19 ± 6 mmHg after 3 months (*p* = 0.002). In the SLT group, IOP decreased from 26 ± 4 mmHg to 18 ± 4 mmHg after 6 weeks and to 15 ± 4 mmHg after 3 months (*p* < 0.001 vs. baseline each). The time course was significantly different between the two groups (*p* = 0.018, ANOVA, Figure 1). In Table 3, mean relative reductions in IOP are shown. While after 6 weeks, no difference between groups was found, after 3 months, the decrease in IOP was significantly more pronounced in the SLT group. After 6 weeks, 11 patients in the ALT group (91.7%) and 12 patients in the SLT group (92.3%) achieved an IOP reduction of >20%. After 3 months, IOP reduction of more than 20% was still present in 9 patients of the ALT group (75%) and 12 patients in the SLT group (92.3%).

Three months after laser treatment, the mean number of IOP-lowering substances used by each patient had decreased in both groups (ALT: from 2.7 ± 0.8 to 2.3 ± 0.9 substances; SLT: from 1.8 ± 1.2 to 1.3 ± 1.1 substances, *p* = 0.386 between groups, ANOVA). The most frequently used substance classes by patients were prostaglandin analogues, followed by beta-receptor antagonists, carbonic anhydrase inhibitors and alpha-2 agonists.

Only a few adverse events were observed. In one patient in the ALT group, episcleritis occurred within 6 weeks after treatment but resolved without sequelae. Two patients in the ALT group and one patient in the SLT group required trabeculectomy within 1 year after laser treatment due to IOP decompensation.

## 4. Discussion

The present study found that both SLT and ALT significantly reduced IOP after 6 weeks and 3 months in patients with POAG, PEX or OHT. After 3 months, the decrease in IOP was significantly more pronounced in the SLT group compared to the ALT group. In addition, 75% of patients in the ALT group had a reduction of more than 20% from baseline IOP, while this was the case in approximately 90% of patients in the SLT group. In both groups, the number of IOP-lowering substances could be reduced after 3 months. Three patients needed trabeculectomy within one year after laser treatment due to IOP compensation: two from the ALT group and one from the SLT group.

The IOP decrease observed in the present study is comparable to reports in the literature, where usually an IOP decrease of 16–30% is seen three months after treatment [9,10,11,12]. In the present study, relative IOP decrease was 26% in the ALT group and 41% in the SLT group after 3 months. A meta-analysis came to similar results after 3 months, finding a significant reduction in IOP in favor of SLT. However, this difference did wear off over time [9]. Another study conducted by Damji et al. found both laser devices to be similarly effective with no difference in terms of efficacy or safety up to 1 year after treatment [13]. In contrast, another meta-analysis found SLT to be more effective in IOP-lowering potential than ALT in general [14]. Since we only followed up patients up to three months regarding IOP, we do not know if this difference would have lasted in our patient cohort.

No difference between the two laser devices in terms of a reduction in used IOP-lowering substances was found, although a tendency towards a decrease was observed. However, the study was not adequately powered to detect these differences, since the primary outcome was the reduction in IOP after laser treatment.

The frequency of AEs was comparable between groups and the rate of patients needing trabeculectomy within one year was very low.

The recently published Laser in Glaucoma and Ocular Hypertension (LiGHT) Trial, which included almost 700 patients and followed most of them over a period of 6 years, also found several advantages of initiating IOP-lowering therapy using SLT instead of topical medication. The SLT group showed better symptom scale scores and better disease control. Additionally, patients initially treated with SLT needed less incisional glaucoma or cataract surgery during the follow-up period compared with patients that started glaucoma treatment using eye drops [15].

The present study has several strengths and limitations. Although it was only a retrospective data analysis, the findings provide real-world data in an office setting with no specific patient selection as is often the case in prospective clinical trials. It is also an advantage that all laser procedures were performed by the same experienced physician, since having different physicians performing the laser procedures can lead to bias [16]. A limitation is the small sample size. However, we could show that even with this relatively small number of patients, the primary endpoint could be reached. The follow-up time was only 3 months, due to the retrospective character of the study, since it is often not possible to obtain data from longer periods and only including long-term data from patients where they are available could have led to bias. Therefore, the present study only provides short-term results and no long-term outcomes. We also were not able to consider diurnal fluctuations of IOP, since these data were not available retrospectively. Still, our results are in good agreement with findings in the literature from both retro- and prospective studies; we therefore think that a similar trend as in other studies, where the treatment effect wears off over time, would be observed if we would have followed up for longer [16,17]. A multi-center study with longer follow-up intervals might be of interest to be conducted in the future.

## 5. Conclusions

In the present study, SLT was at least as effective as ALT with fewer AEs and a similar reduction in concomitant IOP-lowering medication.

## Figures and Tables

**Table 1:** In- and exclusion criteria for selection of patients to be included.

Inclusion Criteria	Exclusion Criteria
Age ≥ 18 years	History of any form of glaucoma surgery
Trabecular meshwork visible for 360 degrees	Use of systemic or topical steroids in the 3 months before ALT/SLT
Primary open-angle glaucoma, pseudoexfoliation glaucoma or ocular hypertension	History of uveitis, acute angle closure or neovascular glaucoma
Received ALT or SLT procedure	Anatomical features not suitable for laser treatment
Follow-up data 6 ± 2 weeks and 3 ± 1 months after surgery were available	

**Table 2:** Baseline data of the two study groups.

	ALT Group (*n* = 12)	SLT Group (*n* = 13)	*p*-Value
**Age (years)**	73 ± 13	72 ± 9	0.993
**Sex (f/m)**	8/4	8/5	0.790
**Glaucoma type**			0.302
**POAG [*n* (%)]**	9 (75.0)	12 (92.3)	
**PEX glaucoma [*n* (%)]**	2 (16.7)	0	
**OHT [*n* (%)]**	1 (8.3)	1 (7.7)	
**IOP at diagnosis (mmHg)**	32 ± 7	29 ± 5	0.152

Values are provided as mean ± SD.

**Table 3:** Relative decrease in IOP of the two study groups at different time points.

	ALT Group (*n* = 12)	SLT Group (*n* = 13)	*p*-Value
**IOP decrease 6 weeks after treatment (%)**	−35 ± 13	−31 ± 9	0.470
**IOP decrease 3 months after treatment (%)**	−26 ± 21	−41 ± 14	0.041

Values are provided as mean ± SD.

## Data Availability

The data presented in this study are available on reasonable request from the corresponding author.
